# From syndromic clues to diagnosis: understanding CARD11-driven disorders

**DOI:** 10.3389/fimmu.2025.1626065

**Published:** 2025-06-23

**Authors:** Elena García-Martínez, María Teresa Schiaffino, Marisa Di Natale, María de las Mercedes Díaz Luna, Daniel Alejandro Viteri Álvarez, María Alejandra Mejía González

**Affiliations:** ^1^ Servicio de Inmunología, Hospital General Universitario Gregorio Marañón, Madrid, Spain; ^2^ Instituto de Investigación Sanitaria Gregorio Marañón (IISGM), Hospital General Universitario Gregorio Marañón., Madrid, Spain

**Keywords:** CARD11, inborn-errors-of-immunity, germline mutations, syndromic immunodeficiencies, BENTA, CADINS

## Abstract

CARD11 is primarily expressed in hematopoietic tissues and lymphocytes and plays a crucial role in the proper activation of B and T cells in response to antigen recognition. Pathogenic variants in the *CARD11* gene result in a broad spectrum of syndromic immunodeficiencies with variable severity and clinical outcomes. Gain-of-function mutations lead to uncontrolled NF-κB activity in lymphocytes and are associated with BENTA syndrome (B-cell Expansion with NF-κB and T-cell Anergy), an autosomal dominant disorder characterized by resistance to conventional therapies used for lymphoproliferative conditions. In contrast, loss-of-function variants are linked to Hyper-IgE-like syndromes, presenting with varying degrees of immunodeficiency—ranging from combined immunodeficiency to specific humoral defects—accompanied by atopic manifestations and autoimmunity. CARD11-associated diseases may be more prevalent than previously recognized due to their clinical overlap with atopic and hematological syndromic disorders. Consequently, a high index of suspicion for these conditions facilitates early diagnosis and enables personalized treatment. In this review, we summarize the broad spectrum of CARD11-related diseases, their underlying pathophysiological mechanisms, multidisciplinary management strategies, and current therapeutic options, along with potential future approaches.

## 1 Introduction

The *CARD11* gene encodes Caspase Recruitment Domain Family Member 11 (CARD11), a large scaffold protein composed of 1,154 amino acids that is predominantly expressed in lymphocytes. This protein plays a pivotal role in the development and proper maturation of B cells and natural killer (NK) cells, and also serves as a central mediator of antigen receptor signaling, orchestrating critical cellular processes such as activation, proliferation, and differentiation of both B and T cells (1).

Structurally, CARD11 is composed of five well-defined domains that underlie its complex regulatory functions: a caspase recruitment domain (CARD), the LATCH domain, a coiled-coil (CC) domain, a centrally located inhibitory domain (ID), and a C-terminal membrane-associated guanylate kinase (MAGUK) domain, which itself comprises PDZ, SH3, and GUK subdomains. In resting lymphocytes, CARD11 is maintained in an inactive conformation through tightly controlled autoinhibitory mechanisms that safeguard against unintended signaling. This basal inactivity is achieved through a network of intramolecular interactions—most notably, hydrophobic contacts between the CARD domain and adjacent linker and coiled-coil regions—that prevent the protein from engaging downstream signaling partners (2). Central to this regulatory architecture is the inhibitory domain (ID), which stabilizes the autoinhibited state. The LATCH domain also plays an essential role by cooperating with the ID to enforce this conformational restraint. Within the ID, four distinct repressor elements (REs) act synergistically to strengthen the autoinhibition, effectively preventing activation under basal conditions (3).

Upon antigen receptor engagement in B or T lymphocytes, CARD11 undergoes a tightly regulated activation process initiated by multisite phosphorylation events, primarily within its inhibitory domain (ID). This post-translational modification is mediated by isoform-specific members of the Protein Kinase C (PKC) family—PKCβ in B cells and PKCθ in T cells—triggering a conformational rearrangement that relieves the protein’s autoinhibitory constraints (4). Additional kinases, such as IKKβ and Akt, have also been implicated in fine-tuning this activation step (5).The resulting structural transition facilitates the recruitment of signaling intermediates and the assembly of the CARD11–BCL10–MALT1 (CBM) complex, a pivotal molecular platform that integrates antigen receptor signals and channels them into downstream effector pathways. Among these, the NF-κB, JNK, and mTOR cascades are prominently activated, promoting lymphocyte survival, proliferation, and the metabolic reprogramming required for full immune competence (6, 7). Together, these molecular events position CARD11 as a critical orchestrator of adaptive immune responses, with a particularly central role in facilitating NF-κB activation following antigen receptor stimulation in both B and T cells.

Given its critical function in lymphocyte biology, germline variants in *CARD11* have been increasingly recognized in patients with a wide range of immune dysregulation syndromes. The clinical manifestations associated with these variants are remarkably diverse and are largely determined by whether the underlying genetic alteration leads to a gain- or loss-of-function (LOF) effect. As novel pathogenic variants continue to emerge, the phenotypic spectrum linked to CARD11 dysfunction continues to expand—ranging from mild, often isolated immune anomalies to complex syndromic presentations—posing both diagnostic challenges and opportunities (8). This work seeks to outline the main phenotypes linked to functional alterations in CARD11, emphasizing the importance for immunology specialists to become familiar with these presentations to enable timely diagnosis and potential therapeutic intervention.

## 2 CARD11 variants: expanding clinical phenotypes in immune dysregulation

### 2.1 Loss-of-function variants in CARD11

Loss-of-function variants in *CARD11* are associated with two distinct types of immunodeficiency according to the latest classification of inborn errors of immunity.

The first is autosomal dominant CARD11 loss-of-function (OMIM: 617638), included within the group of Hyper-IgE syndromes and commonly referred to as CADINS (CARD11-associated atopy with dominant interference of NF-κB signaling). The second is autosomal recessive CARD11 deficiency (OMIM: 615206), which is classified as a form of combined immunodeficiency (CID) (9).

#### 2.1.1 Autosomal dominant CARD11 loss-of-function

CARD11-associated atopy with dominant interference of NF-κB signaling (CADINS) is caused by heterozygous loss-of-function mutations that exert a dominant-negative (DN) effect by disrupting the activity of wild-type CARD11 protein (10). This negative interference is hypothesized to predominantly affect oligomerization-dependent steps of CARD11 activation, with impact on the early conformational transition known as the ‘opening step’ and on the recruitment of signaling cofactors. These stages are critically dependent on the proper assembly of CARD11 oligomers, and disruption by mutant protein subunits is thought to impair signaling efficiency without completely abolishing downstream activity (11). Due to this, most described CADINS-associated variants are hypomorphic and cluster within the N-terminal CARD and LATCH domains, regions essential for these early signaling events. However, dominant-negative mutations have also been reported in other regions, including the GUK subdomain and C-terminal linker regions. Current hypotheses propose that distal variants impair CARD11 function by altering protein folding or conformational stability, thereby hindering effective signal transduction. In addition, C-terminal subdomains have been implicated in the assembly of complex oligomeric structures at the immunological synapse (microclusters), and their disruption may interfere with signalosome architecture, contributing to dominant-negative effects (12–14). Hypomorphic variants in *CARD11*, as found in patients with CADINS, lead to only a partial reduction in protein function, rather than a complete loss. This residual activity allows for some degree of downstream signaling, which contrasts with the profound immunodeficiency seen in individuals carrying biallelic null mutations, where immune function is more severely compromised. In CADINS, the partial disruption of CARD11-mediated signal transduction is sufficient to impair proper immune activation, yet not enough to fully abrogate T cell responses. This subtle imbalance appears to favor the development of Th2-skewed immune profiles, a phenomenon linked to the emergence of atopic and allergic manifestations. A reduction in regulatory T cells has also been observed in some patients, which may further contribute to immune dysregulation (10, 15). Accordingly, the immunodeficiency observed in this context is frequently accompanied by prominent atopic features. Supporting these observations, a heterozygous knock-in mouse model developed by Hutcherson and colleagues replicates the immunological defects expected from these types of CARD11 variants. This model exhibited impaired T cell function, mild B cell abnormalities, reduced regulatory T cell numbers, and elevated serum IgE, although it did not show evidence of a Th2-biased immune response or atopic dermatitis-like symptoms (16).

**Figure 1:**
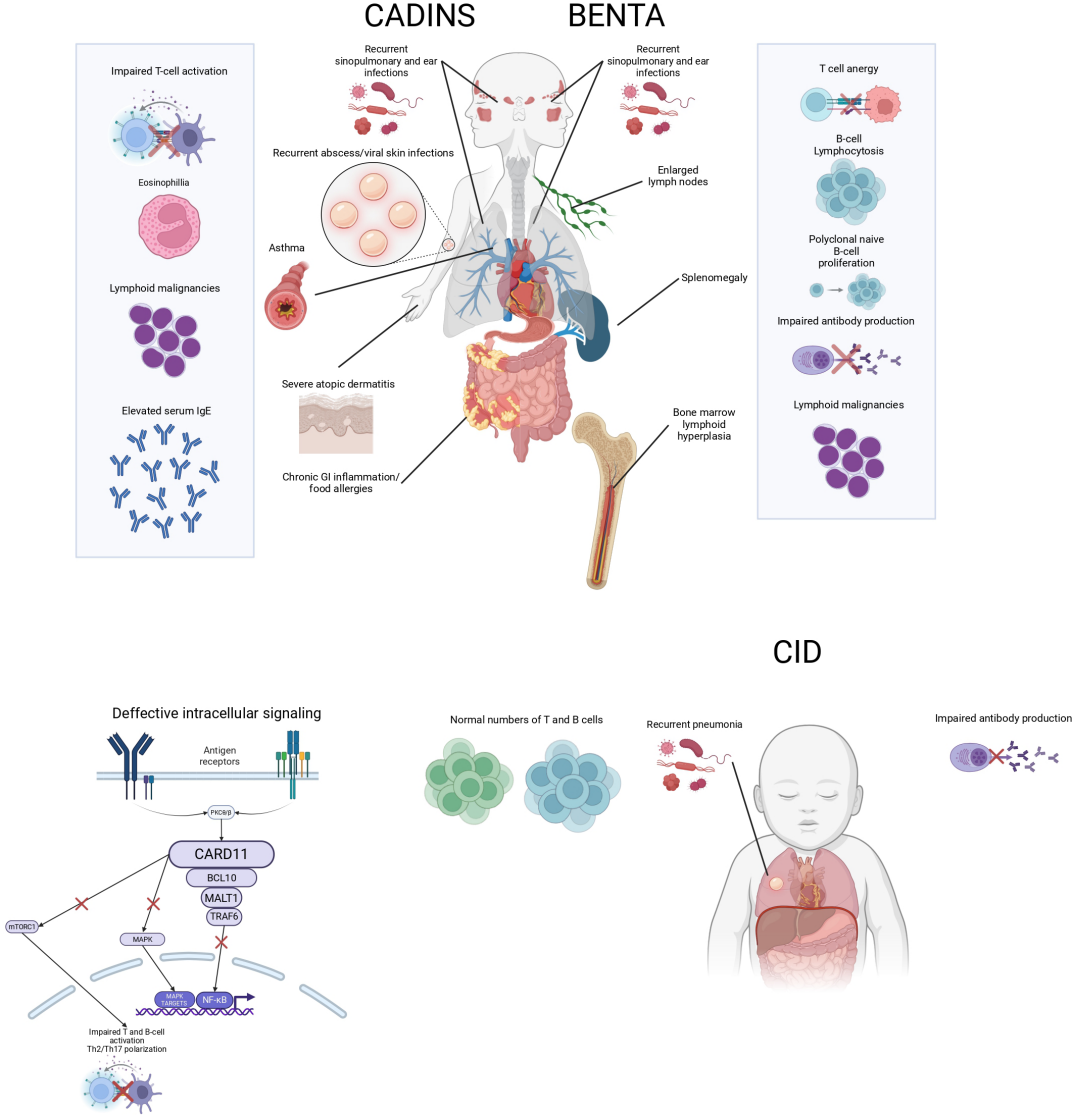
Schematic representation of CARD11-related disorders. The diagram illustrates the clinical features of CADINS, BENTA, and CID, including key immunological abnormalities and associated symptoms.

The degree of immunodeficiency in CADINS is notably variable, with patients exhibiting a wide clinical spectrum that ranges from isolated atopy to more complex immune dysregulation syndromes, including cases of more profound immunodeficiency resembling CID (17). While allergic manifestations are highly prevalent—seen in most individuals carrying confirmed dominant-negative mutations—penetrance is incomplete, and expressivity may differ. Atopic dermatitis is the most frequent allergic phenotype, though other features such as asthma, food allergy and eosinophilic esophagitis can also occur (**Figure 1**). Beyond atopic disease, affected individuals may present with additional immune-related complications, including recurrent skin or respiratory tract infections (chronic sinusitis, pneumonia), autoimmune phenomena, oral ulcers, neutropenia, and impaired B-cell responses (hypogammaglobulinemia, poor antibody responses) (18, 19). Importantly, the absence of atopy does not always preclude CARD11 as a potential disease-causing gene in individuals exhibiting other components of the phenotype (20).

Despite an expanding number of reported variants, clear genotype–phenotype correlations remain elusive, likely due to the influence of genetic modifiers and environmental exposures. Not all monoallelic LOF mutations exhibit strong dominant-negative activity, and in some cases, additional genetic or environmental factors may be necessary to trigger disease manifestations. Indeed, heterozygous LOF variants without dominant-negative effect have been reported in healthy individuals, including carrier parents of patients with biallelic null mutations, suggesting that haploinsufficiency alone may be insufficient to cause disease (7, 15, 21).

#### 2.1.2 Autosomal recessive CARD11 deficiency

Homozygous pathogenic variants in *CARD11* that abolish gene function lead to a severe immunological defect involving both T and B lymphocytes, consistent with a form of profound combined immunodeficiency (**Figure 1**). This autosomal recessive condition is considerably rarer than its dominant counterpart, with only a limited number of cases reported to date. The underlying mutations are predominantly protein-truncating—such as nonsense or frameshift variants—that result in early stop codons or deletions of critical exonic regions, ultimately producing a nonfunctional or absent CARD11 protein (22). However, cases involving homozygous missense mutations have also been described, some presenting with features of both CID and atopic disease, and even one case with inflammatory skin disease without evident CID (23, 24). These observations suggest a scenario in which CARD11 function is severely compromised but not entirely lost, contrasting with the complete deficiency seen in patients with truncating variants.

Given the essential role of CARD11 in lymphocyte differentiation and antigen receptor signaling, complete loss of function disrupts fundamental immune processes from early ontogeny, as also observed in CARD11-deficient mice (1, 25–28). Consequently, clinical presentation often occurs during infancy and includes life-threatening infections, gastrointestinal involvement, and features that may resemble Omenn syndrome, such as a high percentage of naïve T cells and impaired T and B cell activation; or in other cases, lymphadenopathy and dermatitis (erythroderma) (24, 29). The severity and early onset of symptoms underscore the critical requirement for intact CARD11-mediated signaling in maintaining immune homeostasis.

Of note, biallelic deficiencies in the other two components of the CBM complex - BCL10 and MALT1 - share similar clinical phenotypes and are classified within the same group of combined immunodeficiencies. Although only a few cases have been reported for each, they present with early-onset immune dysregulation, including defective lymphocyte activation and proliferation, panhypogammaglobulinemia, recurrent infections, and gastrointestinal symptoms - findings also reproduced in their corresponding experimental mouse models. Nonetheless, due to the broader tissue distribution of BCL10 and MALT1 relative to CARD11, and their ability to interact with other CARD family proteins, additional non-immune manifestations have been observed, such as impaired fibroblast activation in BCL10 deficiency and developmental defects with characteristic facial features in MALT1 deficiency (6).

### 2.2 Gain-of-function variants in CARD11

Gain-of-function (GOF) mutations in *CARD11* are associated with B cell Expansion with NF-κB and T cell Anergy, BENTA syndrome (OMIM: 616452), a rare primary immunodeficiency disorder included within the group of predominantly antibody deficiencies (30).

#### 2.2.1 BENTA syndrome

BENTA syndrome arises from heterozygous germline GOF mutations in *CARD11* that cause constitutive activation of the NF-κB signaling pathway. In B cells, this leads to ligand-independent survival and proliferation, while in T cells, paradoxically, NF-κB persistent activation induces a state of cell hyporesponsiveness characterized by reduced IL-2 production and limited proliferative capacity—an immunological state resembling T cell anergy (31–33).

The underlying molecular mechanism involves disruption of the tightly regulated autoinhibitory conformation of CARD11. As previously described, CARD11 remains inactive in unstimulated lymphocytes through the coordinated action of several structural domains. These include CARD, LATCH, Coiled-coil, and the Inhibitory Domain (ID), which together mediate intramolecular restraint. Among them, the ID plays a central role by housing four repressor elements that act cooperatively to stabilize the inactive state and prevent spontaneous signaling. Gain-of-function mutations disrupt this regulatory architecture, allowing CARD11 to adopt a partially active conformation that aberrantly recruits components of the CBM complex, thereby promoting constitutive NF-κB activation in the absence of receptor engagement (31). Highly active GOF mutations can enhance basal CARD11 signaling activity by up to 160-fold, primarily by overriding the inhibitory constraints of the ID. Interestingly, mutations occurring directly within the ID itself are uncommon, possibly because multiple inhibitory elements would need to be simultaneously disabled to achieve comparable dysregulation (3, 34). Experimental murine models carrying GOF mutations in CARD11, such as E134G, have been instrumental in elucidating BENTA pathogenesis. These mice recapitulate key features of the disease, including accumulation of transitional B cells and impaired antibody responses, and have revealed that CARD11 may also contribute to B cell dysregulation through noncanonical mechanisms beyond NF-κB hyperactivation, such as AKT pathway overactivation and FOXO1 suppression (27).

From a clinical standpoint, BENTA syndrome typically presents with early-onset polyclonal B cell lymphocytosis, characterized by an expansion of naïve and transitional B cells in the peripheral blood. Affected individuals frequently suffer from recurrent sinopulmonary infections and opportunistic viral infections, including molluscum contagiosum, Epstein–Barr virus (EBV), and BK virus. Additional immune abnormalities include impaired antibody responses to polysaccharide antigens—such as pneumococcal and meningococcal vaccines—and a T cell phenotype consistent with anergy (35) (**Figure 1**).

To date, approximately 29 individuals with genetically confirmed BENTA syndrome have been described in the literature (36). Although absolute B lymphocytosis is considered a hallmark, it may not be present in all cases, partly because B cell expansion tends to decline with age and may no longer be apparent in older children or adults (6). Moreover, the presence of increased double-negative T cells, autoimmunity, and lymphoproliferative disease can sometimes mimic an autoimmune lymphoproliferative syndrome (ALPS)-like phenotype (37). A notable and potentially life-threatening complication reported in at least six cases of BENTA syndrome is hemophagocytic lymphohistiocytosis (HLH), which may even represent the initial clinical manifestation in some individuals (30).

#### 2.2.2 Somatic variants and cancer

Several mutations associated with BENTA disease—such as C49Y, G123S, and G123D—have also been found as somatic gain-of-function alterations in Diffuse Large B Cell Lymphoma (DLBCL), pointing to a shared mechanism of NF-κB hyperactivation. This overlap suggests that certain molecular alterations in CARD11 can cause abnormal immune activation in both inherited and acquired conditions (38, 39). In BENTA, germline GOF mutations often lead to chronic polyclonal B cell expansion and immune dysregulation, which may not progress to malignancy. However, in some cases, malignancy can develop in the presence of additional genetic hits or other contributing factors. In contrast, in the somatic context of DLBCL, the same GOF *CARD11* mutations can participate in the process of clonal transformation and lymphoproliferative malignancy. Nevertheless, despite the involvement of shared molecular intermediates, the clinical and cellular consequences of these mutations diverge significantly depending on whether they arise in a malignant or non-malignant setting (31).

## 3 Therapeutic management and outcome

Therapeutic approaches must be individualized based on the specific immunological and clinical phenotype.

### 3.1 Autosomal dominant CARD11 loss-of-function

Heterozygous hypomorphic mutations in *CARD11* can lead to severe atopic disease, associated with weaker, Th2-skewed T cell responses.

Topical corticosteroids and calcineurin inhibitors are standard dermatologic treatments.

Treatment with Dupilumab (40, 41) and omalizumab (41), may offer therapeutic benefit for patients with severe, refractory atopic disease.

Glutamine supplementation has been explored as a treatment that may ameliorate disease in atopic patients harboring functional mutations in *CARD11* by restoring normal glutamine uptake and mTORC1 signaling in activated T cells (10).

### 3.2 Autosomal recessive CARD11 deficiency

CARD11-deficient patients have a profound combined immunodeficiency; therefore, the principal treatment goal should be rapid and definitive immune reconstitution with allogeneic hematopoietic stemcell transplantation. As a bridge to transplantation, patients should receive immunoglobulin replacement and antibiotic prophylaxis for *Pneumocystis jirovecii* (42). The restricted expression of CARD11 in the hematopoietic system makes patients with CARD11 deficiency excellent candidates for stem cell transplantation (43, 44). Functional assays performed in transplanted patients demonstrated that allogeneic HSCT normalizes immune signaling defects and is a definitive treatment for patients with CARD11 deficiency (16).

Similarly, hematopoietic stem cell transplantation is considered the main therapeutic option for complete deficiencies of BCL10 and MALT1, the other two members of the CBM complex. However, evidence remains limited. Only one patient with biallelic BCL10 deficiency has been reported to date and died in early childhood, making treatment outcomes difficult to evaluate. In contrast, hematopoietic stem cell transplantation has been successful in several reported cases of MALT1 deficiency (6).

Although autologous gene therapy has demonstrated success in other primary immunodeficiencies, such as ADA-SCID and Wiskott-Aldrich syndrome, its application in CARD11-related deficiencies has not yet been reported. This may be due to the complex role of CARD11 in immune signaling and the need for precise regulation of its expression to avoid adverse effects.

### 3.3 BENTA syndrome

Sirolimus, an mTOR inhibitor, has been reported as an effective treatment in three patients, leading to a reduction in lymphadenopathy, splenomegaly (8, 45), lymphoid tissue proliferation, and the number of peripheral blood B cells (46). mTOR inhibition has been demonstrated to suppress germinal center responses in peripheral B cells, emphasizing its potential therapeutic utility in refractory B-cell lymphomas. Consequently, sirolimus, is expected to be effective in patients with BENTA (46).

Immunoglobulin replacement therapy is indicated for patients with recurrent infections and hypogammaglobulinemia or impaired vaccine responses. Corticosteroids and rituximab have also been employed in the management of autoimmune complications, particularly autoimmune cytopenias (8).

Given the central role of constitutive NF-κB activation in BENTA disease pathogenesis, investigational agents targeting key components of this pathway are under exploration. Inhibitors of MALT1 protease have emerged as promising candidates, as they modulate CBM complex signaling without inducing complete NF-κB blockade (47), potentially offering a more selective therapeutic approach. Further research is warranted to evaluate the efficacy and safety of these targeted therapies in patients with BENTA.

Optimal management of patients with CARD11 associated diseases requires a multidisciplinary approach that integrates the expertise of multiple medical specialties: clinical immunologist, medical geneticists, dermatologist, hematologists and oncologists and Infectious disease specialists. This collaborative model enhances clinical outcomes and significantly improves long-term quality of life for affected patients.

## 4 Discussion

CARD11 is a scaffold protein essential for immune cell signaling, and its structural integrity is crucial for maintaining immune homeostasis. Mutations affecting CARD11 disrupt this function, leading to immune dysregulation with early-onset and severe manifestations. Despite the clinical heterogeneity that complicates a definitive phenotypic classification, rapid and accurate diagnosis is essential to guide prognosis, therapeutic decision-making, and appropriate monitoring.

Standard newborn screening using T-cell receptor excision circles (TRECs) quantification is currently not effective in detecting CARD11 loss-of-function deficiencies. Although these mutations can cause severe combined immunodeficiencies, TREC levels may remain within normal ranges, making early identification difficult (30–42). To improve the detection of these immunodeficiencies, the implementation of next-generation sequencing (NGS) in neonatal screening programs has been proposed. These technologies could allow for the identification of mutations in genes such as CARD11 before clinical manifestations arise, enabling early intervention and improving patient outcomes (48).

Early recognition of BENTA syndrome, is equally critical. Prompt diagnosis allows for timely treatment of autoimmune manifestations, which may be disease-modifying, and facilitates close monitoring for potentially life-threatening complications such as hemophagocytic lymphohistiocytosis.

Furthermore, maintaining a high index of clinical suspicion and promoting multidisciplinary collaboration among specialists, with immunologists playing a central role, is essential for the accurate evaluation and management of these patients.

In summary, integrating advanced genomic tools and coordinated clinical approaches is crucial for improving diagnosis and care of patients with CARD11 associated diseases.
